# Immunological responses against bovine viral diarrhoea virus types 1 and 2 after administration of a commercial subunit vaccine measured by ELISA and serum neutralisation on serum and milk samples

**DOI:** 10.1002/vro2.70006

**Published:** 2025-03-06

**Authors:** Carlos Montbrau, Marta Gibert, Ester Taberner, Mariona Tapiolas, Raquel Teixeira, Antoni Prenafeta

**Affiliations:** ^1^ Hipra Scientific S.L.U. Amer Spain; ^2^ Hipra Amer Spain

**Keywords:** antibody response, bulk tank milk, BVDV, diagnostic, DIVA, ELISA, marker, neutralising antibodies, vaccine

## Abstract

**Background:**

Our study evaluated the antibody responses against bovine viral diarrhoea virus (BVDV)‐1 and BVDV‐2 generated by a novel subunit vaccine comprising BVDV‐1 E2 and BVDV‐2 E2 recombinant glycoproteins.

**Methods:**

Two trials were conducted. Cattle were given four intramuscular doses (D0, D21, D204 and D570) of 2 mL of vaccine (vaccinated) or phosphate‐buffered saline (control). In the first trial, 20 young calves were randomly distributed into the vaccinated or control group. Blood samples were collected to assess BVDV antibodies by ELISA and neutralisation. In the second trial, heifers and cows from three farms were assigned to the vaccinated and control groups. Individual blood samples, bulk tank milk (BTM) and individual milk samples were obtained. The antibody response against BVDV was analysed using different ELISA kits. Neutralising antibodies against different BVDV isolates from Europe and North and South America were also assessed in this trial.

**Results:**

Compared with the control group, the vaccine induced significantly (*p* < 0.05) higher levels of antibodies against BVDV‐1 and BVDV‐2 from 21 days after the second dose until the end of the study. In the second trial, high levels of total antibodies against BVDV were observed, with no induction of anti‐p80 antibodies observed in vaccinated animals or in individual samples (milk and sera) or BTM samples. High levels of neutralising antibodies were observed against different BVDV isolates from Europe and America.

**Conclusions:**

The vaccine induced a strong antibody response against BVDV‐1 and BVDV‐2; this response allowed infected and vaccinated animals to be differentiated (Differentiating Infected from Vaccinated Animals (DIVA) vaccine).

## INTRODUCTION

Bovine viral diarrhoea (BVD) is recognised as one of the most important endemic diseases in cattle with a significant economic impact on the industry. This bovine pestivirus is distributed worldwide with only a few European countries having eradicated the virus.^1^ Bovine viral diarrhoea virus (BVDV) infection is usually not noticed by farmers, except in the case of rare virulent outbreaks. In most cases, the clinical presentation is non‐specific and limited to a few days of fever and loss of appetite. Once a herd is exposed to BVDV, reproductive problems caused by the disease can occur, including transient infertility, abortion, stillbirths, malformed calves and persistently infected (PI) calves. Other manifestations of the disease on herd health can also be detected, including milk production and clinical mastitis, with an increase in respiratory and enteric diseases.^2^

Various BVD control programmes around the world have been developed for the prevention and control of the disease through vaccination. Vaccination against BVDV is an effective tool for disease control because it reduces the risk of reinfection in the herd,^3^ as well as the likelihood of fetal infection.^4^ The use of BVDV vaccines raises concerns about potential interference in relation to induced antibody responses, impairing the interpretation of serological surveillance in the herd.^5^ The use of laboratory diagnostic assays to detect BVDV‐specific antibodies in individual samples of serum and milk, as well as in bulk tank milk (BTM), is often part of BVD control programmes in terms of both serological surveillance and estimating BVDV prevalence in the herd. Detection of the virus (antigen ELISA or real‐time reverse transcriptase polymerase chain reaction [RT‐PCR]) is used to identify PI animals in order to cull them and to reduce the risk of infection within the herd.^6^

The structural proteins of BVDV are the nucleocapsid and the three envelope glycoproteins E^rns^, E1 and E2.^7^ The structural envelope glycoprotein E2 is the major immunogenic determinant of the BVDV virion.^8^ Neutralising (SN) antibodies induced in infected animals are mainly directed against E2.^9^ Moreover, E2‐specific monoclonal antibodies can neutralise both BVDV‐1 (pestivirus A) and BVDV‐2 (pestivirus B).^10^

The non‐structural catalytic serine protease (NS3 or p80) is a highly immunogenic protein from BVDV and is the basis of several commercially available ELISAs. Some authors have reported that after the primary course of inactivated BVDV vaccines, antibodies against the p80 protein were not induced.^11^ Other studies^3^, ^12^, ^13^, ^14^ have reported inconclusive results regarding the reliability of different commercial p80 ELISAs to distinguish vaccinated from infected animals.

A novel BVDV subunit vaccine (DIVENCE)^15^ comprising different bovine respiratory and reproductive antigens, including BVDV‐1 E2 and BVDV‐2 E2 recombinant glycoproteins, has been developed. The efficacy of DIVENCE has been reported in experimental challenges of BVDV‐1 and BVDV‐2 in pregnant animals.^16^ The immune response of those animals was assessed, with high titres of total antibodies (ELISA) and SN antibodies against BVDV‐1 and BVDV‐2 being described on the day of challenge. Challenge trials cannot be conducted for all potential infection situations, such as days after vaccination or days of gestation. Furthermore, animal welfare concerns prevent their widespread use. Consequently, antibody analysis is regarded as a useful method to assess the immune responses induced by the vaccines.^8^

The design of the E2‐recombinant protein‐based BVDV vaccine allows infected animals to be differentiated from vaccinated animals. Such DIVA, or marker, vaccines have been used in the veterinary field for decades.^17^ They carry at least one antigenic protein less than the wildtype virus; the diagnostic test thus measures the antibodies against the absent protein(s) to facilitate identification of infected animals.^17^

The purpose of this study was to determine the immune response against BVDV‐1 and BVDV‐2 induced by DIVENCE, as measured by different assays on serum and milk samples after vaccination; also to demonstrate the capacity of the vaccine to differentiate between vaccinated and infected animals.

## MATERIALS AND METHODS

### Trial 1

#### Animals and housing

Twenty Friesian‐Holstein heifer calves (55‒104 days of age) were selected from commercial farms in Catalonia (Northeast Spain). The farms had no known recent history of BVDV and had administered no previous vaccination against this disease. The calves were serologically screened for BVDV antibodies (BVDV Total Antibody Test, IDEXX) and were confirmed to be free of persistent BVDV infection by real‐time RT‐qPCR before inclusion in the study. The group size was calculated to achieve significant differences between groups, complying with the European Pharmacopeia requirements for safety and efficacy trials,^18^, ^19^ also to ensure monitoring of the immune response for almost 2 years. The group size was determined to contain at least eight animals during the entire study.

Twenty heifer calves were randomly divided into two study groups of 10 animals (vaccinated and control groups) and individually identified. The randomisation was conducted by sorting all the animals by their age (days of life); then, 10 ranks of two animals were generated following this order. A random number was assigned using Microsoft Excel. Each animal with a lower random number per rank was assigned to the control group; the other animal was assigned to the vaccinated group. The entire study was conducted on a commercial farm and was carried out on a blinded basis. Both groups were housed together and treated as the same batch of animals. The staff involved in the animal experimental phase were not aware of the group of each individual animal.

All calf‐handling practices were approved by the local authority (Generalitat de Catalunya) and followed the recommendations of Directive 2010/63/EU of the European Parliament (https://eur‐lex.europa.eu/eli/dir/2010/63/oj/eng) and of the Council on the protection of animals used for scientific purposes as well as the HIPRA Animal Experimentation Committee. All animals used in this study were handled in strict accordance with good clinical practice and all efforts were made to minimise suffering. This phase was conducted over a 20‐month period.

#### Vaccination and study design

All animals received a 2 mL intramuscular dose of DIVENCE or phosphate‐buffered saline (PBS) according to the group. The vaccine was reconstituted and administered according to the manufacturer's instructions.

Two doses were administered 3 weeks apart (D0 and D21 of the trial) for the basic vaccination scheme. Six months later, a booster was administered (D204); a further year later, subsequent re‐vaccination (D570) was performed. During the entire study, from the first vaccination dose until 21 days after the fourth dose, all animals were checked daily by a technician and weekly by a veterinarian to assess safety parameters, including depression and systemic reactions. Blood samples were collected from all animals to assess total antibodies against BVDV (ELISA) and SN antibodies against BVDV‐1 and BVDV‐2. These blood samples were collected prior to vaccination (D0; first dose V1) and on days 21 (D21; second dose V2), 42, 154, 204 (D204; third dose V3), 225, 280, 395, 497, 570 (D570; fourth dose V4) and 591 of the study. Whole blood samples were also collected prior to vaccination (D0) to obtain buffy coats in order to assess the absence of BVDV virus before the study began.

#### Laboratory analysis

All serum samples were analysed using a commercial ELISA (BVDV Total Antibody Test, IDEXX; ELISA A). This ELISA is based on the use of antigens derived from whole BVDV. Serum samples were also analysed using a neutralisation test in MDBK cells in 96‐well plates to quantitate SN antibodies against BVDV‐1 and BVDV‐2. Singer (BVDV‐1) and VV‐670 (BVDV‐2) cytopathic virus strains were used to perform the neutralisation test. A constant viral titre (10^2^ CCID_50_) was incubated with twofold dilutions of sera. Culture plates were incubated for 7 days and visually assessed for virus‐induced cytopathic effects.

### Trial 2

#### Animals and study design

Three commercial dairy farms from Spain were selected (Son is the farm Son Carbó (Campos, Mallorca, Spain), SUB is the farm Subaida (Es Mercadal, Menorca, Spain and TON Can Toni Roure (Maçanet de la Selva, Girona, Spain)), which were all seronegative for BVDV at the start of the study. For each herd, a sample of BTM before vaccination was collected and analysed by PCR and ELISA (CIVTEST Bovis BVD/BD p80; HIPRA) by an external laboratory, Associació Interprofessional Lletera de Catalunya; all farms had a negative result.

The study was designed as a multicentre, randomised, placebo‐controlled, double‐blind field trial comparing the DIVENCE vaccine to a placebo (PBS). On each farm, heifers were randomly allocated into two groups on D0, the DIVENCE or control group, stratified by animal category (heifer or cow). In the heifer category, animals from 10 months of age until calving were included, and in the cow category, animals between first and seventh calvings were included (Table 1). The randomisation was carried out separately in the seven stratified categories: heifers less than 12 months, heifers 12 months or more, cows in the first, second, third and fourth lactations, or with more than four lactations. In each category, a random number was assigned to each animal using Microsoft Excel. Around half of the animals were vaccinated with DIVENCE and the other half received PBS (SON: 260 control and 258 vaccinated animals; SUB: 73 control and 75 vaccinated animals; TON: 191 control and 198 vaccinated animals), using the same volume and the same days described for trial 1. The animal distribution is detailed in Table 1.

**TABLE 1:** Description of study animal distribution three farms and groups in relation to age and the number of lactations.

		Farm SON		Farm SUB		Farm TON	
		Control	Vaccine	Control	Vaccine	Control	Vaccine
Heifer	≥12 months	67	61	22	22	31	32
	<12 months	51	51	8	10	17	19
	Total	118	112	30	32	48	51
Cow	1st lactation	50	60	13	16	67	67
	2nd lactation	35	35	14	11	40	45
	3rd lactation	28	24	9	10	21	22
	4th lactation	13	11	3	3	7	9
	>4th lactation	16	16	4	3	8	4
	Total	142	146	43	43	143	147
Total		260	258	73	75	191	198

Blood samples were collected from 32 random animals on each farm (16 vaccinated and 16 control animals) immediately prior to vaccination (D0) and on days 21, 42, 204, 225, 385, 570 and 591 of the study. Additionally, BTM samples from each farm were obtained prior to vaccination and approximately 12, 15, 18, 21 and 24 months after the first dose of vaccine (D0). Additionally, on farm TON, individual milk samples were collected from 58 milking cows (27 vaccinated animals and 31 control) on D591 (21 days after the fourth vaccination).

The trial was conducted in compliance with the Good Clinical Practice Guidance Document.^20^

#### Laboratory analysis

Three different commercial ELISA tests were used in this trial. The BVDV Total Antibody Test (IDEXX) is based on use of antigens derived from whole BVDV (ELISA A, total antibodies). The other two ELISA tests were specifically based on detection of the p80 antigen (BVDV NS3) of the virus: CIVTEST Bovis BVD/BD p80 (HIPRA; ELISA B) and BVDV p80 antibody test (IDEXX; ELISA C). All these ELISAs were conducted in accordance with the manufacturer's instructions.

All the serum samples were analysed with ELISAs A and B. The serum samples from one farm (SON) were also analysed with ELISA C. The BTM samples were analysed using these three different commercial ELISAs A, B and C. Individual milk samples were analysed using ELISAs A and B.

Additionally, serum samples from five vaccinated animals from one farm (TON) collected on D591 of the study (21 days after the fourth dose; V4) were analysed to determine SN antibodies. For this purpose, BVDV serum SN antibody detection in MDBK cells in 96‐well plates was used to quantitate SN antibodies against BVDV‐1 and BVDV‐2. Several virus strains (from Europe and North and South America) were used for this, including Singer (BVDV‐1a), SKOL 1146 (BVDV‐1a), Phillips P3 (BVDV‐1b), RVB1297 (BVDV‐1d) and SDARS 91 W, Iguazú, Ghent, VV‐670 (all BVDV‐2). A constant virus titre (10^2^ CCID_50_) was incubated with twofold dilutions of sera. The culture plates were incubated for 7 days and visually assessed for a virus‐induced cytopathic effect in the case of cytopathic strains. For non‐cytopathic strains, culture plates were incubated for 4 days and an immunoperoxidase monolayer assay was performed.

### Statistical analysis

Statistical analyses and plots were generated using R (version 4.0.5, 2021, R Core Team; R: A Language and Environment for Statistical Computing, R Foundation for Statistical Computing) and Microsoft Excel 2010 (Microsoft Corporation). All the figures presented values from the raw data. For the analysis of longitudinal data (titres of total antibodies against BVDVD and p80 antibodies by ELISA in serum), mixed effects models of repeated measures (MMRM) were fitted using gls() function from the nlme R package, assuming normality of the errors. For the assessment of the significance of each term in fixed effects part, while controlling for other predictors, a marginal analysis of variance, that is, type III sum of squares ANOVA, was conducted. In these models, the group factor, the day factor and the group‐by‐day interaction were included as fixed effects. Days of age at first vaccination was included in these models as covariate. An unstructured variance‒covariance matrix was employed to model within‐subject errors. If convergence issues were encountered a first‐order autoregressive variance‒covariance matrix was modelled instead (sequential approach until the model convergence). Models for SN antibody (against BVDV‐1 and BVDV‐2) data of trial 1 were fitted for the vaccinated groups only from day 21 because the other group and time conditions had all observations equal to zero. Restricted (residual) maximum likelihood (REML) was used for model estimation and multiple comparisons were adjusted using the multivariate *t*‐distribution correction in the emmeans package of R. Total antibodies and anti‐p80 antibodies were compared using a Mann‒Whitney *U*‐test between groups of individual milk samples and using an ANOVA test on BTM samples. A significant level of *p*‐value less than 0.05 was used for all variables evaluated in this trial.

## RESULTS

### Trial 1

**FIGURE 1:**
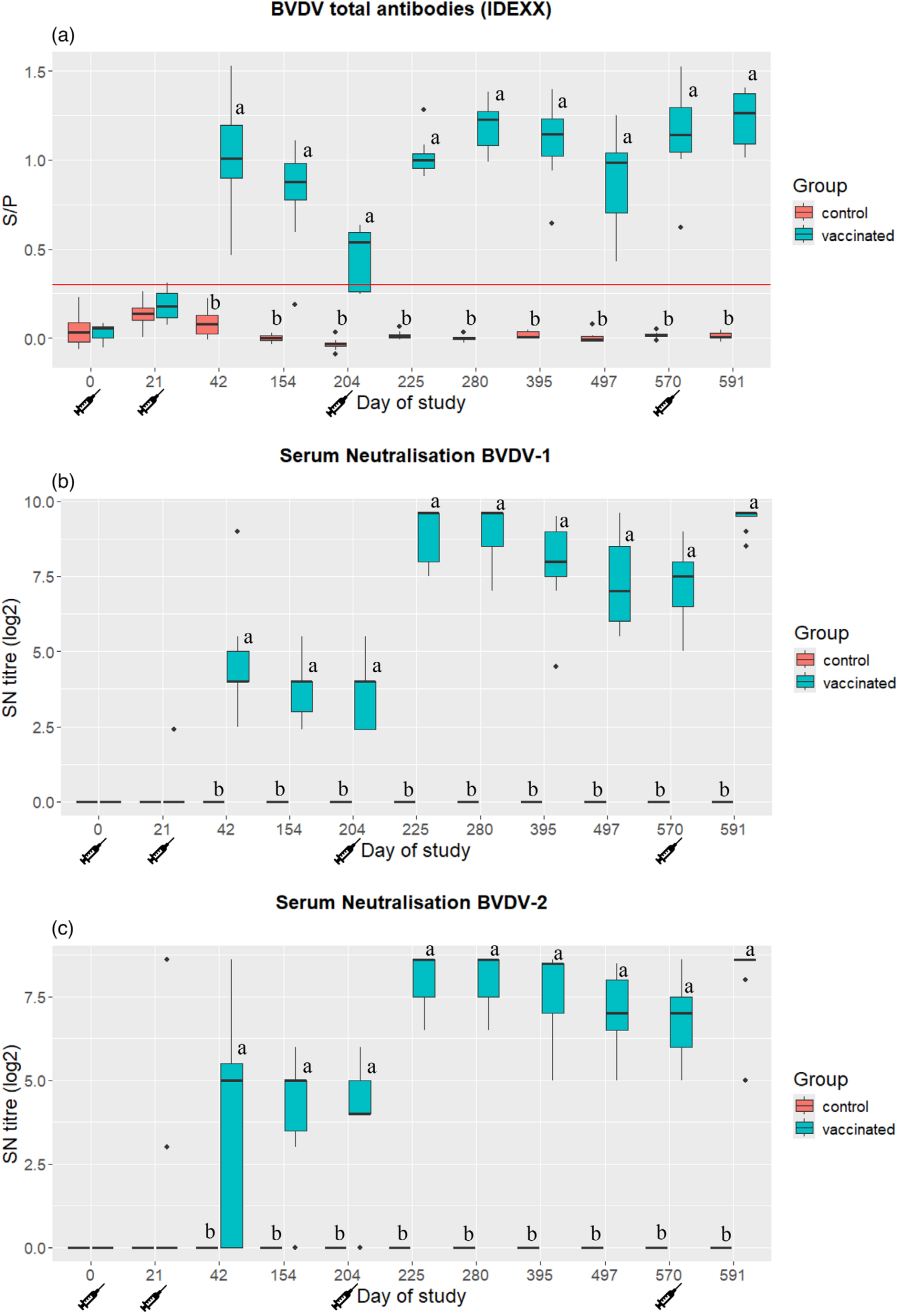
Antibody titres results for serum samples tested by ELISA and neutralising (SN) antibodies from first vaccination (D0 of study) to 21 days after the 1‐year booster vaccination (D594 of study). (A) Sample/positive control index (S/P) measured using a total antibody ELISA (BVDV Total Antibody Test, IDEXX); red line marks the cut‐off (S/P values greater than 0.3 were considered positive). (B) Neutralising antibodies against bovine viral diarrhoea virus (BVDV)‐1 using Singer strain. (C) Neutralising antibodies against BVDV‐2 using VV‐670 strain. Letters (a and b) indicate statistically significant differences (*p* < 0.05) between antibody titres between the control and vaccinated groups. Box plots with median value and first and third quartile values.

Mean serum ELISA values for total antibody detection and SN antibodies against BVDV‐1 (Singer) and BVDV‐2 (VV670) for vaccinated and control animals are shown in Figure 1 (A, B and C, respectively; statistical results are detailed in Tables S1 and S2). Compared to the control group, the vaccinated animals showed a significantly (*p* < 0.05) higher titre of total and SN antibodies against BVDV‐1 and BVDV‐2 from day 42 post‐vaccination until the end of the study.

After the primary vaccination (two doses of DIVENCE), the antibody titre, as detected by ELISA and SN antibodies against BVDV‐1 and BVDV‐2 in the vaccinated animals, remained similar from D42 until the administration of the third dose (D204). After the third dose, a clear boost in antibody response was then induced in terms of both total and SN antibodies against BVDV‐1 and BVDV‐2. The high titres remained stable during the entire year after the third dose, from D225 to D570. All vaccinated animals had SN antibodies against BVDV‐1 from D42 until the end of the study. In terms of SN antibodies against BVDV‐2, all animals had antibodies during the entire year after the third dose.

### Trial 2

#### Serology

**FIGURE 2:**
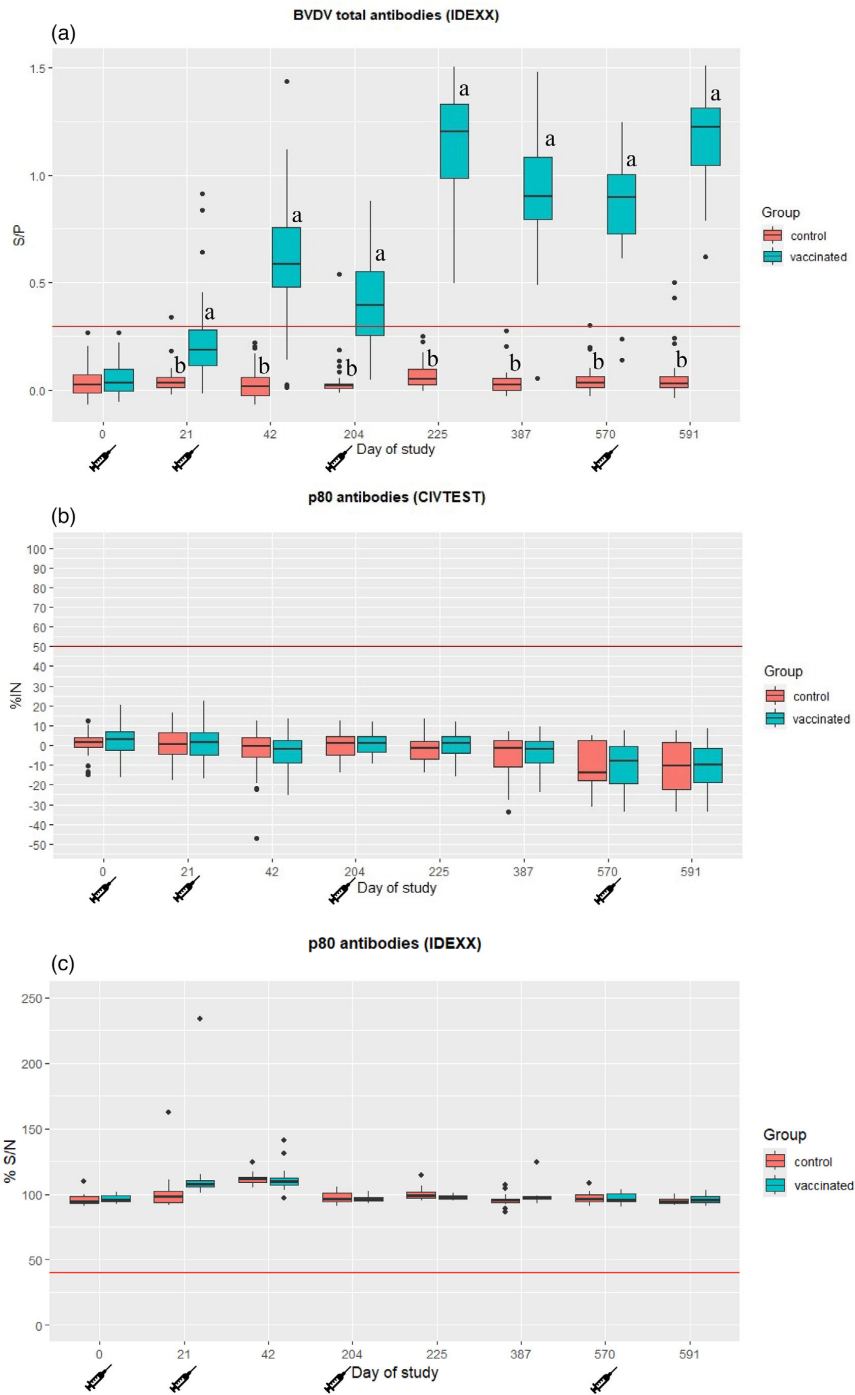
ELISA results from serum samples tested using three different kits. (A) Sample/positive control index (S/P) measured using a total antibody ELISA (BVDV Total Antibody Test, IDEXX); red line marks the cut‐off (S/P values greater than 0.3 were considered positive). (B) Inhibition percentage (%IN) value measured using a p80 antibody ELISA (CIVTEST Bovis BVD/BD p80; red line marks the cut‐off (%IN values greater than 50 were considered positive). (C) Signal/noise percentage (%S/N) for p80 antibody (IDEXX BVDV p80 antibody); red line marks the cut‐off (%S/N values lower than 40 were considered positive). Letters (a and b) indicate statistically significant differences between groups (*p* < 0.05). Box plots with median value and first and third quartile values. BVDV, bovine viral diarrhoea virus.

Mean serum ELISA values for total antibody detection and anti‐p80 antibody detection for vaccinated and control animals are shown in Figure 2 (statistical results are detailed in Tables S3, S4 and S5). Vaccinated animals showed a significant (*p* < 0.05) increase in total antibodies at day 21 post‐vaccination. Peak mean total antibodies were reached on D225 of the study, 21 days after the third dose of DIVENCE. Antibody levels remained high over the entire year after the third dose, then decreased slightly prior to the fourth dose of the vaccine (D570). In terms of the proportion of animals with antibodies, the vast majority had antibodies against BVDV during this period from the third dose to the fourth dose (Figure 2). Three out of 48 control animals on one of the days had positive results for total antibodies, as detected by ELISA, during the entire study. Of these, none had SN antibodies against BVDV‐1 or BVDV‐2. Consequently, vaccinated animals had a significantly (*p* < 0.05) higher proportion of seropositive animals compared to the control group from D21 to the end of the study (D591).

In terms of anti‐p80 antibodies (ELISAs B and C), all the animals in the vaccinated and control groups remained negative during the entire study; consequently, no differences between groups were observed.

**FIGURE 3:**
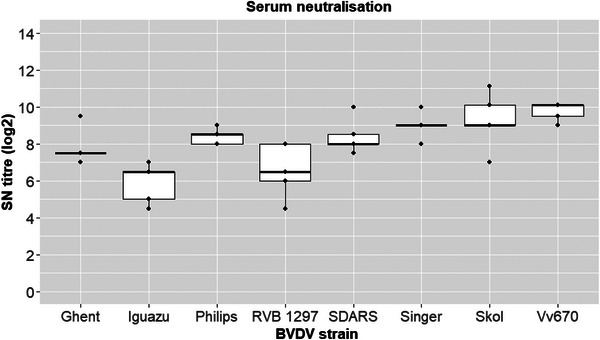
Neutralising antibodies against several strains of bovine viral diarrhoea virus (BVDV)‐1 and BVDV‐2 measured by a serum neutralisation test at day 591 of study. Box plots with median value and first and third quartile values.

Neutralising antibodies against several strains (BVDV‐1: Singer [BVDV‐1a], SKOL 1146 [BVDV‐1a], Phillips P3 [BVDV‐1b], RVB1297 [BVDV‐1d]; BVDV‐2: SDARS 91 W, Iguazú, Ghent, VV‐670) were analysed from serum samples collected from five vaccinated animals on day 591 of the study (Figure 3). All blood samples had SN antibodies against all strains listed. Mean SN antibody titres in these vaccinated animals were log_2_ 7.7 (range log_2_ 4‒11.1) against BVDV‐1 strains and log_2_ 8.0 (range log_2_ 4.5‒10.1) against BVDV‐2 strains.

#### Milk samples

**FIGURE 4:**
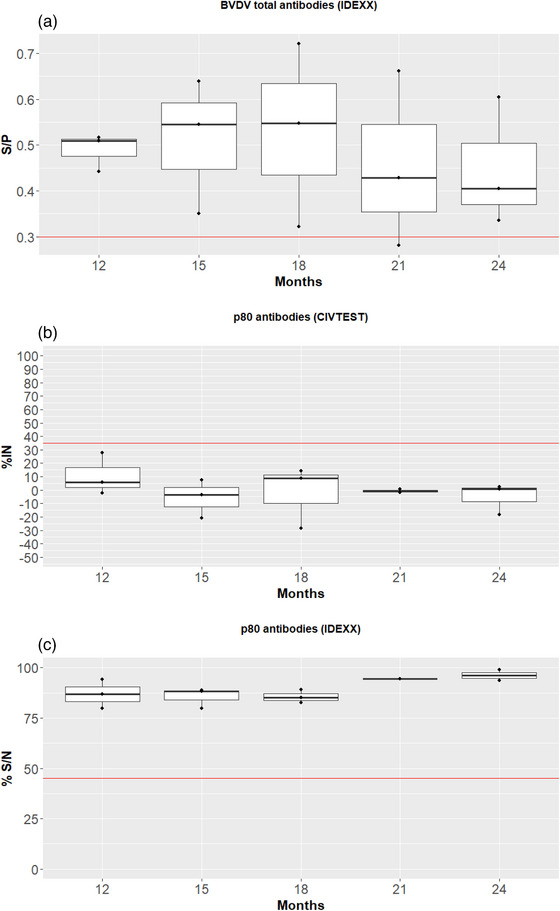
ELISA results from bulk tank milk samples tested using three different kits. (A) Sample/positive control index (S/P) measured using a total antibody ELISA (BVDV Total Antibody Test, IDEXX); red line marks the cut‐off (S/P values greater than 0.3 were considered positive). (B) Inhibition percentage (%IN) value measured using a p80 antibody ELISA (CIVTEST Bovis BVD/BD p80); red line marks the cut‐off (%IN values greater than 35 were considered positive). (C) Signal/noise percentage (%S/N) for p80 antibody (IDEXX BVDV p80 antibody); red line marks the cut‐off (%S/N values lower than 45 were considered positive). Box plots with median value and first and third quartile values. BVDV, bovine viral diarrhoea virus.

Before vaccination, all BTM samples were negative by ELISA (CIVTEST Bovis BVD/BD p80; HIPRA). After the first vaccination, total and anti‐p80 antibodies were determined in BTM samples with ELISAs A, B and C. No statistical differences were observed between sampling points after vaccination (12, 15, 18, 21 and 24 months after the first vaccination; Figure 4) for total and anti‐p80 antibodies.

In terms of total antibodies against BVDV (ELISA A), the vast majority of samples had antibodies against BVDV for 24 months after vaccination. Conversely, all BTM samples were negative for anti‐p80 antibodies (ELISAs B and C) from 12 to 24 months after vaccination.

**FIGURE 5:**
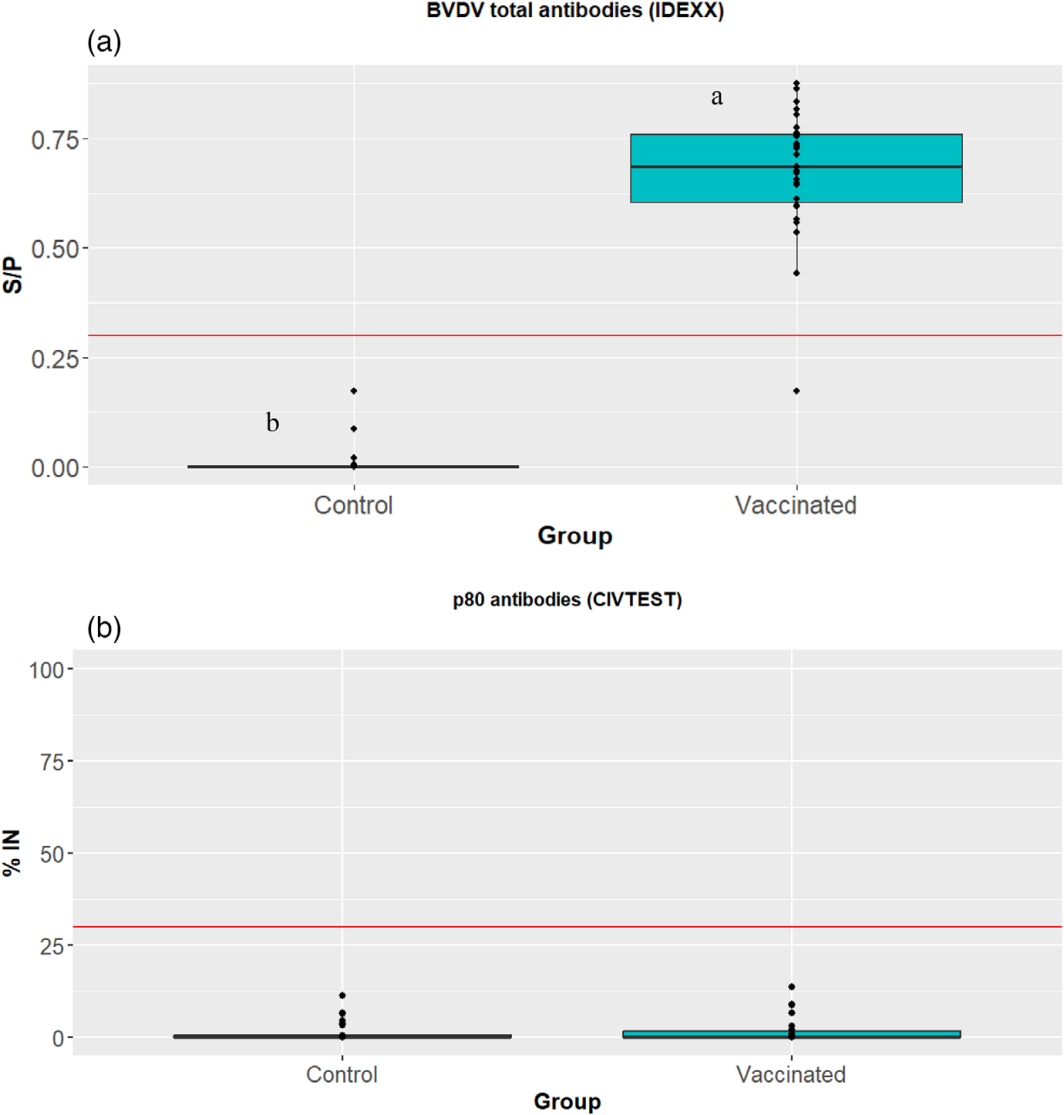
ELISA results from individual milk samples from vaccinated and control animals at day 591 of the study, tested using two different kits. (A) Sample/positive control index (S/P) measured using a total antibody ELISA (BVDV Total Antibody Test, IDEXX), red line marks the cut‐off (S/P values greater than 0.3 were considered positive). (B) Inhibition percentage (%IN) value measured using a p80 antibody ELISA (CIVTEST Bovis BVD/BD p80), red line marks the cut‐off (%IN values greater than 30 were considered positive). Letters (a and b) indicate statistically significant differences between groups (*p* < 0.05). Box plots with median value and first and third quartile values. BVDV, bovine viral diarrhoea virus.

Individual milk samples collected 21 days after the fourth vaccination were analysed using ELISAs A and B. In terms of total antibodies against BVDV, 26 out of the 27 vaccinated animals were seropositive, while all control animals were seronegative. Consequently, vaccinated animals had significantly (*p* < 0.05) higher total antibody titres against BVDV compared to control animals (Figure 5). At the same time, anti‐p80 antibody levels remained negative in both groups; no statistically significant differences were observed between control and vaccinated animals.

## DISCUSSION

The aim of this study was to investigate the immune response against BVDV‐1 and BVDV‐2 induced by the DIVENCE vaccine and to assess its suitability as a DIVA vaccine.

In the first trial, the vaccine resulted in a clear increase in antibodies, both in terms of ELISA (total antibodies) and SN antibodies. The immune response remained stable for 12 months after the third dose, with no statistical differences found between sampling points (from D225 to D570 of the study). Given the characteristics of dairy farms, with animals at different production stages across the year, it must be ensured that all animals achieve a good immunological response which remains high throughout the production cycle. If infection with non‐cytopathic BVDV strains occurs during the first 125 days of gestation, the fetus can become PI with the virus.^21^ Therefore, a high and sustained response over time is fundamental as a tool to fight against transplacental BVDV infection.

The efficacy of DIVENCE against transplacental infection by BVDV‐1 and BVDV‐2 in pregnant animals has been reported.^16^ In that study, DIVENCE was found to induce both a marked humoral and cellular immune response, increasing the level of antibodies and interferon gamma.^16^ The results obtained in that study on SN antibody titres in vaccinated animals on the day of both challenges were statistically similar to the levels obtained in the present study at all sampling points from D225 to D570. These results show that the immune response induced by DIVENCE after the re‐vaccination scheme confers high and stable levels of SN antibodies against BVDV‐1 and BVDV‐2 for 1 year.

In the second trial, the humoral response of DIVENCE was evaluated on serum and milk samples (BTM and individual) with different ELISA kits, one based on the use of antigens derived from whole BVDV (ELISA A) and two specifically based on anti‐p80 antibodies (BVDV NS3, ELISAs B and C). The vaccine induced a strong increase in total antibody levels from day 21 of the study, with negative anti‐p80 antibody titres in serum and milk samples, even after subsequent re‐vaccinations. In terms of milk samples, only one vaccinated animal had no anti‐BVDV antibodies in the milk, equivalent to 3.7% of the vaccinated animals. This may be due to different causes, such as the sensitivity of the IDEXX Total Antibody Test (96.3%), the individual response which may be affected by the state of the animal the day it received the fourth dose, or influences such as high milk production, which could dilute the antibody titre in the milk. In any case, it should be noted that, while vaccination efficacy may depend on the individual (animal), it is effective at the herd level.^8^, ^21^, ^22^ The use of safer vaccines such as DIVENCE allows mass vaccination strategies, which have been demonstrated to increase the success of vaccination programmes by enhancing herd immunity.^21^, ^22^

The structural envelope glycoprotein E2, is the major immunogenic determinant of the BVDV virion.^8^ Neutralising antibodies induced in infected animals are mainly directed against E2.^9^ Moreover, monoclonal antibodies specific to E2 have demonstrated a virus‐neutralising ability for both BVDV‐1 and BVDV‐2.^10^ These findings support the design of a BVDV vaccine based on E2 to distinguish infected from vaccinated animals. Accordingly, the E2 protein has been suggested as a promising approach for a subunit vaccine.^23^ Several studies have reported on this approach to future vaccine candidates.^24^, ^25^, ^26^, ^27^, ^28^, ^29^, ^30^

Since non‐structural proteins are mainly produced during viral replication, cattle develop antibodies to these antigens following natural infection and/or vaccination with modified live‐virus (MLV) vaccines. The interference of MLV vaccines in diagnostic tests goes beyond antibody detection. The strains contained in MLV vaccines have been found to be detectable by PCR in ear notch samples of newborn calves,^31^ requiring additional analysis to differentiate infected from vaccinated calves. Consequently, inactivated BVDV vaccines, where the virus cannot replicate, have been suggested to have an advantage over MLV vaccines in that they may not induce detectable antibodies against the BVDV p80 protein. However, the use of the antibodies against p80 to monitor BVDV circulation in herds after vaccination with inactivated vaccines has failed; false‐positive results were often obtained and the use of inactivated vaccines has been shown to interfere with ELISA monitoring in blood and milk samples.^3^, ^12^, ^13^, ^14^

The subunit vaccine described here induces antibodies against the E2 protein only, brings the characteristic of a real DIVA vaccine and solves these limitations.

The DIVENCE vaccine induced SN antibodies against different BVDV strains. Four strains from BVDV‐1 and four strains from BVDV‐2 from countries in Europe and North and South America were selected to represent the antigenic diversity of this virus worldwide. Serum from all vaccinated animals was found to neutralise all strains tested.

A comparison of the SN antibodies titres obtained in this study with those induced by other vaccines was out of the scope of this trial. As tests are not standardised between laboratories, a reliable comparison of this kind would be difficult. The technique depends on the cell system and the strain of virus used.^32^ However, a comparison between the SN antibody titres obtained in this trial and those obtained in previous trials using the same standardised test is possible. The SN antibody levels achieved against all strains tested were higher than those obtained against the Iguazú strain, which is the challenge strain used in a previous study.^16^

DIVENCE combines the immunogenicity of live‐attenuated vaccines with the safety of inactivated vaccines. This vaccine provided a strong and sustained immune response against the most prevalent subgenotypes of BVDV‐1 and BVDV‐2 (cross‐protection). Compared to ordinary BVDV vaccines, DIVENCE also allows infected animals and vaccinated animals to be differentiated (DIVA, or marker vaccine), making it a new tool to control and monitor the BVDV status of farms.

## AUTHOR CONTRIBUTIONS

Carlos Montbrau, Marta Gibert, Antoni Prenafeta and Ester Taberner conceived and designed the project. Carlos Montbrau, Marta Gibert, Ester Taberner and Mariona Tapiolas acquired the data. Carlos Montbrau, Marta Gibert, Ester Taberner, Mariona Tapiolas and Raquel Teixeira analysed and interpreted the data. Carlos Montbrau, Marta Gibert, Ester Taberner, Mariona Tapiolas and Raquel Teixeira wrote the paper.

## CONFLICTS OF INTEREST

The authors declare they have no conflicts of interest.

## ETHICS STATEMENT

This trial was approved by the Spanish National Agency for Medicines and Medical Devices (AEMPS; license no. 21/010).

## Supporting information



Supporting information

## Data Availability

Research data are not shared.
