# Supplementary material for: Immunological responses against bovine viral diarrhoea virus types 1 and 2 after administration of a commercial subunit vaccine measured by ELISA and serum neutralisation on serum and milk samples
Source: Vet Rec Open. 2025 Mar 6;12(1):e70006. doi: 10.1002/vro2.70006 (PMC11883183; doi:10.1002/vro2.70006)
Supplement: Supplementary file 1 — Supporting information [file VRO2-12-e70006-s001.docx]

# Supporting Information

Statistical results obtained from all model parameters.

## TABLE S1 Summary of the statistical results obtained on Trial 1 from total antibodies against BVDV data

| **Model ANOVA table (Type III Sum of squares)** | | | |
| --- | --- | --- | --- |
|  | Denominator degree of freedom | | 188 |
|  | Number of degrees of Freedom | F-Value | p-value |
| Intercept | 1 | 463.33 | <0.0001 |
| Group | 1 | 298.14 | <0.0001 |
| Day | 10 | 258.72 | <0.0001 |
| Age0 | 1 | 313.86 | <0.0001 |
| Group:Day | 10 | 357.08 | <0.0001 |

| **BVDV Total Antibodies (IDEXX)** | | | | | | |
| --- | --- | --- | --- | --- | --- | --- |
| Time point |  | Statistic |  | Control |  | Vaccine |
| D0 |  | Number of animals with observations |  | 10 |  | 10 |
|  |  | Adjusted treatment mean |  | 0.045 |  | 0.035 |
|  |  | Standard error |  | 0.029 |  | 0.016 |
|  |  | 95% confidence interval |  | -0.011, 0.102 |  | 0.003, 0.066 |
|  |  | Adjusted treatment mean difference control versus vaccine | | |  |  |
|  |  | Difference |  | 0.0105 |  |  |
|  |  | Standard error |  | 0.0326 |  |  |
|  |  | p-value |  | 0.7492 |  |  |
| D21 |  | Number of animals with observations |  | 10 |  | 10 |
|  |  | Adjusted treatment mean |  | 0.127 |  | 0.186 |
|  |  | Standard error |  | 0.025 |  | 0.027 |
|  |  | 95% confidence interval |  | 0.077,0.177 |  | 0.131,0.240 |
|  |  | Adjusted treatment mean difference control versus vaccine | | |  |  |
|  |  | Difference |  | -0.0582 |  |  |
|  |  | Standard error |  | 0.0373 |  |  |
|  |  | p-value |  | 0.1213 |  |  |
| D42 |  | Number of animals with observations |  | 10 |  | 9 |
|  |  | Adjusted treatment mean |  | 0.081 |  | 0.993 |
|  |  | Standard error |  | 0.026 |  | 0.089 |
|  |  | 95% confidence interval |  | 0.030, 0.132 |  | 0.816, 1.170 |
|  |  | Adjusted treatment mean difference control versus vaccine | | |  |  |
|  |  | Difference |  | -0.9117 |  |  |
|  |  | Standard error |  | 0.0931 |  |  |
|  |  | p-value |  | <0.0001 |  |  |
| D154 |  | Number of animals with observations |  | 10 |  | 9 |
|  |  | Adjusted treatment mean |  | -0.003 |  | 0.822 |
|  |  | Standard error |  | 0.004 |  | 0.085 |
|  |  | 95% confidence interval |  | -0.010, 0.005 |  | 0.653, 0.991 |
|  |  | Adjusted treatment mean difference control versus vaccine | | |  |  |
|  |  | Difference |  | -0.8246 |  |  |
|  |  | Standard error |  | 0.0852 |  |  |
|  |  | p-value |  | <0.0001 |  |  |

## TABLE S1 (continuation)

| **BVDV Total Antibodies (IDEXX)** | | | | | | |
| --- | --- | --- | --- | --- | --- | --- |
| Time point |  | Statistic |  | Control |  | Vaccine |
| D204 |  | Number of animals with observations |  | 10 |  | 9 |
|  |  | Adjusted treatment mean |  | -0.036 |  | 0.461 |
|  |  | Standard error |  | 0.011 |  | 0.072 |
|  |  | 95% confidence interval |  | -0.058, -0.013 |  | 0.318, 0.604 |
|  |  | Adjusted treatment mean difference control versus vaccine | | |  |  |
|  |  | Difference |  | -0.4963 |  |  |
|  |  | Standard error |  | 0.0731 |  |  |
|  |  | p-value |  | <0.0001 |  |  |
| D225 |  | Number of animals with observations |  | 10 |  | 9 |
|  |  | Adjusted treatment mean |  | 0.014 |  | 1.017 |
|  |  | Standard error |  | 0.008 |  | 0.080 |
|  |  | 95% confidence interval |  | -0.001, 0.029 |  | 0.859, 1.175 |
|  |  | Adjusted treatment mean difference control versus vaccine | | |  |  |
|  |  | Difference |  | -1.0025 |  |  |
|  |  | Standard error |  | 0.0802 |  |  |
|  |  | p-value |  | <0.0001 |  |  |
| D280 |  | Number of animals with observations |  | 10 |  | 9 |
|  |  | Adjusted treatment mean |  | -0.003 |  | 1.187 |
|  |  | Standard error |  | 0.011 |  | 0.035 |
|  |  | 95% confidence interval |  | -0.025, 0.019 |  | 1.118, 1.257 |
|  |  | Adjusted treatment mean difference control versus vaccine | | |  |  |
|  |  | Difference |  | -1.1904 |  |  |
|  |  | Standard error |  | 0.0370 |  |  |
|  |  | p-value |  | <0.0001 |  |  |
| D395 |  | Number of animals with observations |  | 10 |  | 9 |
|  |  | Adjusted treatment mean |  | 0.014 |  | 1.091 |
|  |  | Standard error |  | 0.011 |  | 0.064 |
|  |  | 95% confidence interval |  | -0.009, 0.036 |  | 0.964, 1.218 |
|  |  | Adjusted treatment mean difference control versus vaccine | | |  |  |
|  |  | Difference |  | -1.0770 |  |  |
|  |  | Standard error |  | 0.0650 |  |  |
|  |  | p-value |  | <0.0001 |  |  |
| D497 |  | Number of animals with observations |  | 10 |  | 9 |
|  |  | Adjusted treatment mean |  | 0.000 |  | 0.889 |
|  |  | Standard error |  | 0.019 |  | 0.067 |
|  |  | 95% confidence interval |  | -0.038, 0.038 |  | 0.757, 1.022 |
|  |  | Adjusted treatment mean difference control versus vaccine | | |  |  |
|  |  | Difference |  | -0.8890 |  |  |
|  |  | Standard error |  | 0.0695 |  |  |
|  |  | p-value |  | <0.0001 |  |  |

## TABLE S1 (continuation)

| **BVDV Total Antibodies (IDEXX)** | | | | | | |
| --- | --- | --- | --- | --- | --- | --- |
| Time point |  | Statistic |  | Control |  | Vaccine |
| D570 |  | Number of animals with observations |  | 10 |  | 9 |
|  |  | Adjusted treatment mean |  | 0.013 |  | 1.139 |
|  |  | Standard error |  | 0.008 |  | 0.073 |
|  |  | 95% confidence interval |  | -0.002, 0.028 |  | 0.994, 1.284 |
|  |  | Adjusted treatment mean difference control versus vaccine | | |  |  |
|  |  | Difference |  | -1.1260 |  |  |
|  |  | Standard error |  | 0.0737 |  |  |
|  |  | p-value |  | <0.0001 |  |  |
| D591 |  | Number of animals with observations |  | 10 |  | 9 |
|  |  | Adjusted treatment mean |  | 0.010 |  | 1.236 |
|  |  | Standard error |  | 0.006 |  | 0.136 |
|  |  | 95% confidence interval |  | -0.002, 0.021 |  | 0.967, 1.505 |
|  |  | Adjusted treatment mean difference control versus vaccine | | |  |  |
|  |  | Difference |  | -1.2268 |  |  |
|  |  | Standard error |  | 0.1358 |  |  |
|  |  | p-value |  | <0.0001 |  |  |

## TABLE S2 Summary of the statistical results obtained on Trial 1 from neutralizing antibodies against BVDV-1 and BVDV-2 data

**Model ANOVA table (Type III Sum of squares) SN against BVDV-1**

|  | Denominator degree of freedom | | 80 |
| --- | --- | --- | --- |
|  | Number of degrees of freedom | F-Value | p-value |
| Intercept | 1 | 108.99 | <.0001 |
| Day | 9 | 143.36 | <.0001 |
| Age0 | 1 | 0.5309 | 0.4683 |

**Model ANOVA table (Type III Sum of squares) SN against BVDV-2**

|  | Denominator degree of freedom | | 80 |
| --- | --- | --- | --- |
|  | Number of degrees of freedom | F-Value | p-value |
| Intercept | 1 | 89.473 | <.0001 |
| Day | 9 | 14.859 | <.0001 |
| Age0 | 1 | 11.105 | 0.0013 |

| **Serum Neutralisation Vaccinated group** | | | | | | |
| --- | --- | --- | --- | --- | --- | --- |
| Time point |  | Statistic |  | BVDV-1 |  | BVDV-2 |
| D21 |  | Number of animals with observations |  | 10 |  | 10 |
|  |  | Adjusted treatment mean |  | 0.228 |  | 1.079 |
|  |  | Standard error |  | 0.299 |  | 0.876 |
|  |  | 95% confidence interval |  | -0.447, 0.903 |  | -0.892, 3.050 |
|  |  | Adjusted treatment mean difference vaccine versus 0 | | |  |  |
|  |  | p-value |  | 0.986 |  | 0.847 |
| D42 |  | Number of animals with observations |  | 9 |  | 9 |
|  |  | Adjusted treatment mean |  | 4.616 |  | 3.627 |
|  |  | Standard error |  | 0.753 |  | 1.378 |
|  |  | 95% confidence interval |  | 2.843, 6.387 |  | 0.353, 6.900 |
|  |  | Adjusted treatment mean difference vaccine versus 0 | | |  |  |
|  |  | p-value |  | 0.0034 |  | 0.1989 |
| D154 |  | Number of animals with observations |  | 9 |  | 9 |
|  |  | Adjusted treatment mean |  | 3.701 |  | 4.073 |
|  |  | Standard error |  | 0.304 |  | 0.747 |
|  |  | 95% confidence interval |  | 3.030,4.372 |  | 2.290,5.855 |
|  |  | Adjusted treatment mean difference vaccine versus 0 | | |  |  |
|  |  | p-value |  | <0.0001 |  | 0.0107 |
| D204 |  | Number of animals with observations |  | 9 |  | 9 |
|  |  | Adjusted treatment mean |  | 3.561 |  | 4.158 |
|  |  | Standard error |  | 0.380 |  | 0.577 |
|  |  | 95% confidence interval |  | 2.724,4.396 |  | 2.856,5.458 |
|  |  | Adjusted treatment mean difference vaccine versus 0 | | |  |  |
|  |  | p-value |  | <0.0001 |  | 0.0004 |

## TABLE S2 (continuation)

| **Serum Neutralisation Vaccinated group** | | | | | | |
| --- | --- | --- | --- | --- | --- | --- |
| Time point |  | Statistic |  | BVDV-1 |  | BVDV-2 |
| D225 |  | Number of animals with observations |  | 9 |  | 9 |
|  |  | Adjusted treatment mean |  | 8.943 |  | 7.949 |
|  |  | Standard error |  | 0.272 |  | 0.291 |
|  |  | 95% confidence interval |  | 8.341, 9.544 |  | 7.292, 8.606 |
|  |  | Adjusted treatment mean difference vaccine versus 0 | | |  |  |
|  |  | p-value |  | <0.0001 |  | <0.0001 |
| D280 |  | Number of animals with observations |  | 9 |  | 9 |
|  |  | Adjusted treatment mean |  | 8.944 |  | 8.075 |
|  |  | Standard error |  | 0.304 |  | 0.198 |
|  |  | 95% confidence interval |  | 8.279, 9.608 |  | 7.644, 8.505 |
|  |  | Adjusted treatment mean difference vaccine versus 0 | | |  |  |
|  |  | p-value |  | <0.0001 |  | <0.0001 |
| D395 |  | Number of animals with observations |  | 9 |  | 9 |
|  |  | Adjusted treatment mean |  | 7.889 |  | 7.697 |
|  |  | Standard error |  | 0.445 |  | 0.323 |
|  |  | 95% confidence interval |  | 6.909, 8.868 |  | 6.993, 8.401 |
|  |  | Adjusted treatment mean difference vaccine versus 0 | | |  |  |
|  |  | p-value |  | <0.0001 |  | <0.0001 |
| D497 |  | Number of animals with observations |  | 9 |  | 9 |
|  |  | Adjusted treatment mean |  | 7.401 |  | 7.065 |
|  |  | Standard error |  | 0.392 |  | 0.273 |
|  |  | 95% confidence interval |  | 6.544, 8.256 |  | 6.458, 7.671 |
|  |  | Adjusted treatment mean difference vaccine versus 0 | | |  |  |
|  |  | p-value |  | <0.0001 |  | <0.0001 |
| D570 |  | Number of animals with observations |  | 9 |  | 9 |
|  |  | Adjusted treatment mean |  | 7.279 |  | 6.854 |
|  |  | Standard error |  | 0.401 |  | 0.394 |
|  |  | 95% confidence interval |  | 6.382, 8.175 |  | 5.975, 7.731 |
|  |  | Adjusted treatment mean difference vaccine versus 0 | | |  |  |
|  |  | p-value |  | <0.0001 |  | <0.0001 |
| D591 |  | Number of animals with observations |  | 9 |  | 9 |
|  |  | Adjusted treatment mean |  | 9.401 |  | 8.143 |
|  |  | Standard error |  | 0.147 |  | 0.354 |
|  |  | 95% confidence interval |  | 9.051, 9.751 |  | 7.343, 8.942 |
|  |  | Adjusted treatment mean difference vaccine versus 0 | | |  |  |
|  |  | p-value |  | <0.0001 |  | <0.0001 |

## TABLE S3 Summary of the statistical results obtained on Trial 2 from total antibodies against BVDV data from individual serum samples

| **Model ANOVA table (Type III Sum of squares)** | | | |
| --- | --- | --- | --- |
|  | Denominator degree of freedom | | 650 |
|  | Number Degrees of Freedom | F-Value | p-value |
| Intercept | 1 | 495.24 | <.0001 |
| Group | 1 | 601.31 | <.0001 |
| Day | 7 | 179.81 | <.0001 |
| Age0 | 1 | 0.4224 | 0.5160 |
| Lactation | 1 | 0.0467 | 0.8291 |
| Group:Day | 7 | 165.22 | <.0001 |

| **BVDV Total Antibodies (IDEXX)** | | | | | | |
| --- | --- | --- | --- | --- | --- | --- |
| Time point |  | Statistic |  | Control* |  | Vaccine* |
| D0 |  | Number of animals with observations |  | 47 |  | 47 |
|  |  | Adjusted treatment mean |  | 0.040 |  | 0.054 |
|  |  | Standard error |  | 0.009 |  | 0.011 |
|  |  | 95% confidence interval |  | 0.022, 0.058 |  | 0.031, 0.076 |
|  |  | Adjusted treatment mean difference control versus vaccine | | |  |  |
|  |  | Difference |  | -0.014 |  |  |
|  |  | Standard error |  | 0.015 |  |  |
|  |  | p-value |  | 0.339 |  |  |
| D21 |  | Number of animals with observations |  | 47 |  | 47 |
|  |  | Adjusted treatment mean |  | 0.045 |  | 0.227 |
|  |  | Standard error |  | 0.008 |  | 0.031 |
|  |  | 95% confidence interval |  | 0.029, 0.060 |  | 0.166, 0.287 |
|  |  | Adjusted treatment mean difference control versus vaccine | | |  |  |
|  |  | Difference |  | -0.182 |  |  |
|  |  | Standard error |  | 0.032 |  |  |
|  |  | p-value |  | <0.0001 |  |  |
| D42 |  | Number of animals with observations |  | 47 |  | 47 |
|  |  | Adjusted treatment mean |  | 0.035 |  | 0.605 |
|  |  | Standard error |  | 0.011 |  | 0.040 |
|  |  | 95% confidence interval |  | 0.013, 0.057 |  | 0.526, 0.683 |
|  |  | Adjusted treatment mean difference control versus vaccine | | |  |  |
|  |  | Difference |  | -0.570 |  |  |
|  |  | Standard error |  | 0.042 |  |  |
|  |  | p-value |  | <0.0001 |  |  |
| D204 |  | Number of animals with observations |  | 41 |  | 46 |
|  |  | Adjusted treatment mean |  | 0.040 |  | 0.402 |
|  |  | Standard error |  | 0.012 |  | 0.031 |
|  |  | 95% confidence interval |  | 0.015, 0.063 |  | 0.340, 0.462 |
|  |  | Adjusted treatment mean difference control versus vaccine | | |  |  |
|  |  | Difference |  | -0.362 |  |  |
|  |  | Standard error |  | 0.033 |  |  |
|  |  | p-value |  | <0.0001 |  |  |

*The missing samples from day 0 to day 591 correspond to loss to follow-up animals, such as reproductive, low production, lameness, mastitis and accident reasons

## TABLE S3 (continuation)

| **BVDV Total Antibodies (IDEXX)** | | | | | | |
| --- | --- | --- | --- | --- | --- | --- |
| Time point |  | Statistic |  | Control* |  | Vaccine* |
| D225 |  | Number of animals with observations |  | 39 |  | 46 |
|  |  | Adjusted treatment mean |  | 0.069 |  | 1.137 |
|  |  | Standard error |  | 0.010 |  | 0.036 |
|  |  | 95% confidence interval |  | 0.048, 0.088 |  | 1.066, 1.208 |
|  |  | Adjusted treatment mean difference control versus vaccine | | |  |  |
|  |  | Difference |  | -1.069 |  |  |
|  |  | Standard error |  | 0.038 |  |  |
|  |  | p-value |  | <0.0001 |  |  |
| D387 |  | Number of animals with observations |  | 39 |  | 40 |
|  |  | Adjusted treatment mean |  | 0.034 |  | 0.896 |
|  |  | Standard error |  | 0.008 |  | 0.048 |
|  |  | 95% confidence interval |  | 0.018, 0.050 |  | 0.800, 0.990 |
|  |  | Adjusted treatment mean difference control versus vaccine | | |  |  |
|  |  | Difference |  | -0.861 |  |  |
|  |  | Standard error |  | 0.049 |  |  |
|  |  | p-value |  | <0.0001 |  |  |
| D570 |  | Number of animals with observations |  | 32 |  | 35 |
|  |  | Adjusted treatment mean |  | 0.052 |  | 0.845 |
|  |  | Standard error |  | 0.011 |  | 0.039 |
|  |  | 95% confidence interval |  | 0.030, 0.073 |  | 0.767, 0.921 |
|  |  | Adjusted treatment mean difference control versus vaccine | | |  |  |
|  |  | Difference |  | -0.792 |  |  |
|  |  | Standard error |  | 0.041 |  |  |
|  |  | p-value |  | <0.0001 |  |  |
| D591 |  | Number of animals with observations |  | 33 |  | 35 |
|  |  | Adjusted treatment mean |  | 0.061 |  | 1.171 |
|  |  | Standard error |  | 0.017 |  | 0.036 |
|  |  | 95% confidence interval |  | 0.028, 0.094 |  | 1.100, 1.241 |
|  |  | Adjusted treatment mean difference control versus vaccine | | |  |  |
|  |  | Difference |  | -1.110 |  |  |
|  |  | Standard error |  | 0.0397 |  |  |
|  |  | p-value |  | <0.0001 |  |  |

* The missing samples from day 0 to day 591 correspond to loss to follow-up animals, such as reproductive, low production, lameness, mastitis and accident reasons

## TABLE S4 Summary of the statistical results obtained on Trial 2 from p80 antibodies (CIVTEST) data from individual serum samples

| **Model ANOVA table (Type III Sum of squares)** | | | |
| --- | --- | --- | --- |
|  | Denominator degree of freedom | | 651 |
|  | Number of degrees of freedom | F-Value | p-value |
| Intercept | 1 | 6.5360 | 0.0108 |
| Group | 1 | 1.7614 | 0.1849 |
| Day | 7 | 16.685 | <.0001 |
| Age0 | 1 | 0.4410 | 0.5069 |
| Lactation | 1 | 1.9454 | 0.1636 |
| Group:Day | 7 | 0.4999 | 0.8348 |

| **BVDV p80 Antibodies (CIVTEST)** | | | | | | |
| --- | --- | --- | --- | --- | --- | --- |
| Time point |  | Statistic |  | Control* |  | Vaccine* |
| D0 |  | Number of animals with observations |  | 47 |  | 47 |
|  |  | Adjusted treatment mean |  | 1.061 |  | 2.924 |
|  |  | Standard error |  | 0.786 |  | 1.058 |
|  |  | 95% confidence interval |  | -0.482, 2.604 |  | 0.847, 5.000 |
|  |  | Adjusted treatment mean difference control versus vaccine | | |  |  |
|  |  | Difference |  | -1.863 |  |  |
|  |  | Standard error |  | 1.32 |  |  |
|  |  | p-value |  | 0.1581 |  |  |
| D21 |  | Number of animals with observations |  | 47 |  | 47 |
|  |  | Adjusted treatment mean |  | 0.479 |  | 1.327 |
|  |  | Standard error |  | 1.102 |  | 1.155 |
|  |  | 95% confidence interval |  | -1.686, 2.642 |  | -0.942, 3.595 |
|  |  | Adjusted treatment mean difference control versus vaccine | | |  |  |
|  |  | Difference |  | -0.848 |  |  |
|  |  | Standard error |  | 1.60 |  |  |
|  |  | p-value |  | 0.5954 |  |  |
| D42 |  | Number of animals with observations |  | 47 |  | 47 |
|  |  | Adjusted treatment mean |  | -2.696 |  | -2.911 |
|  |  | Standard error |  | 1.592 |  | 1.321 |
|  |  | 95% confidence interval |  | -5.822, 0.429 |  | -5.506, -0.31 |
|  |  | Adjusted treatment mean difference control versus vaccine | | |  |  |
|  |  | Difference |  | 0.215 |  |  |
|  |  | Standard error |  | 2.07 |  |  |
|  |  | p-value |  | 0.9173 |  |  |
| D204 |  | Number of animals with observations |  | 41 |  | 46 |
|  |  | Adjusted treatment mean |  | 0.306 |  | 1.341 |
|  |  | Standard error |  | 0.967 |  | 0.911 |
|  |  | 95% confidence interval |  | -1.592, 2.204 |  | -0.449, 3.130 |
|  |  | Adjusted treatment mean difference control versus vaccine | | |  |  |
|  |  | Difference |  | -1.035 |  |  |
|  |  | Standard error |  | 1.33 |  |  |
|  |  | p-value |  | 0.4370 |  |  |

* The missing samples from day 0 to day 591 correspond to loss to follow-up animals, such as reproductive, low production, lameness, mastitis and accident reasons

## TABLE S4 (continuation)

| **BVDV p80 Antibodies (CIVTEST)** | | | | | | |
| --- | --- | --- | --- | --- | --- | --- |
| Time point |  | Statistic |  | Control* |  | Vaccine* |
| D225 |  | Number of animals with observations |  | 40 |  | 46 |
|  |  | Adjusted treatment mean |  | -1.556 |  | 1.136 |
|  |  | Standard error |  | 0.993 |  | 1.017 |
|  |  | 95% confidence interval |  | -3.507, 0.395 |  | -0.862, 3.133 |
|  |  | Adjusted treatment mean difference control versus vaccine | | |  |  |
|  |  | Difference |  | -2.692 |  |  |
|  |  | Standard error |  | 1.42 |  |  |
|  |  | p-value |  | 0.0590 |  |  |
| D387 |  | Number of animals with observations |  | 39 |  | 40 |
|  |  | Adjusted treatment mean |  | -5.675 |  | -3.139 |
|  |  | Standard error |  | 1.563 |  | 1.211 |
|  |  | 95% confidence interval |  | -8.745, -2.606 |  | -5.518, -0.760 |
|  |  | Adjusted treatment mean difference control versus vaccine | | |  |  |
|  |  | Difference |  | -2.536 |  |  |
|  |  | Standard error |  | 1.98 |  |  |
|  |  | p-value |  | 0.2002 |  |  |
| D570 |  | Number of animals with observations |  | 32 |  | 35 |
|  |  | Adjusted treatment mean |  | -9.724 |  | -8.531 |
|  |  | Standard error |  | 2.027 |  | 1.866 |
|  |  | 95% confidence interval |  | -13.705, -5.744 |  | -12.195, -4.866 |
|  |  | Adjusted treatment mean difference control versus vaccine | | |  |  |
|  |  | Difference |  | -1.193 |  |  |
|  |  | Standard error |  | 2.76 |  |  |
|  |  | p-value |  | 0.6652 |  |  |
| D591 |  | Number of animals with observations |  | 33 |  | 35 |
|  |  | Adjusted treatment mean |  | -10.833 |  | -9.148 |
|  |  | Standard error |  | 2.074 |  | 2.043 |
|  |  | 95% confidence interval |  | -14.906, -6.761 |  | -13.161, -5.136 |
|  |  | Adjusted treatment mean difference control versus vaccine | | |  |  |
|  |  | Difference |  | -1.685 |  |  |
|  |  | Standard error |  | 2.91 |  |  |
|  |  | p-value |  | 0.5628 |  |  |

* The missing samples from day 0 to day 591 correspond to loss to follow-up animals, such as reproductive, low production, lameness, mastitis and accident reasons

## TABLE S5 Summary of the statistical results obtained on Trial 2 from p80 antibodies (IDEXX) data from individual serum samples

| **Model ANOVA table (Type III Sum of squares)** | | | |
| --- | --- | --- | --- |
|  | Denominator degree of freedom | | 226 |
|  | Number of Degrees of Freedom | F-Value | p-value |
| Intercept | 1 | 6676.5 | <.0001 |
| Group | 1 | 0.873 | 0.3512 |
| Day | 7 | 28.386 | <.0001 |
| Age0 | 1 | 2.753 | 0.0985 |
| Lactation | 1 | 3.984 | 0.0471 |
| Group:Day | 7 | 3.180 | 0.0031 |

| **BVDV p80 Antibodies (IDEXX)** | | | | | | |
| --- | --- | --- | --- | --- | --- | --- |
| Time point |  | Statistic |  | Control* |  | Vaccine* |
| D0 |  | Number of animals with observations |  | 16 |  | 16 |
|  |  | Adjusted treatment mean |  | 96.11 |  | 96.54 |
|  |  | Standard error |  | 1.06 |  | 0.96 |
|  |  | 95% confidence interval |  | 94.02, 98.19 |  | 94.64, 98.43 |
|  |  | Adjusted treatment mean difference control versus vaccine | | |  |  |
|  |  | Difference |  | -0.424 |  |  |
|  |  | Standard error |  | 1.43 |  |  |
|  |  | p-value |  | 0.7674 |  |  |
| D21 |  | Number of animals with observations |  | 16 |  | 16 |
|  |  | Adjusted treatment mean |  | 102.95 |  | 115.45 |
|  |  | Standard error |  | 4.87 |  | 7.20 |
|  |  | 95% confidence interval |  | 93.33, 112.5 |  | 101.2, 129.6 |
|  |  | Adjusted treatment mean difference control versus vaccine | | |  |  |
|  |  | Difference |  | -12.506 |  |  |
|  |  | Standard error |  | 8.69 |  |  |
|  |  | p-value |  | 0.1520 |  |  |
| D42 |  | Number of animals with observations |  | 16 |  | 16 |
|  |  | Adjusted treatment mean |  | 112.00 |  | 111.90 |
|  |  | Standard error |  | 1.31 |  | 2.46 |
|  |  | 95% confidence interval |  | 109.4, 114.5 |  | 107.0, 116.7 |
|  |  | Adjusted treatment mean difference control versus vaccine | | |  |  |
|  |  | Difference |  | 0.107 |  |  |
|  |  | Standard error |  | 2.79 |  |  |
|  |  | p-value |  | 0.9695 |  |  |
| D204 |  | Number of animals with observations |  | 16 |  | 16 |
|  |  | Adjusted treatment mean |  | 97.96 |  | 96.73 |
|  |  | Standard error |  | 1.13 |  | 0.67 |
|  |  | 95% confidence interval |  | 95.73, 100.1 |  | 95.41, 98.05 |
|  |  | Adjusted treatment mean difference control versus vaccine | | |  |  |
|  |  | Difference |  | 1.226 |  |  |
|  |  | Standard error |  | 1.31 |  |  |
|  |  | p-value |  | 0.3523 |  |  |

* The missing samples from day 0 to day 591 correspond to loss to follow-up animals, such as reproductive, low production, lameness, mastitis and accident reasons.

## TABLE S5 (continuation)

| **BVDV p80 Antibodies (IDEXX)** | | | | | | |
| --- | --- | --- | --- | --- | --- | --- |
| Time point |  | Statistic |  | Control* |  | Vaccine* |
| D225 |  | Number of animals with observations |  | 15 |  | 16 |
|  |  | Adjusted treatment mean |  | 100.63 |  | 97.71 |
|  |  | Standard error |  | 1.25 |  | 0.50 |
|  |  | 95% confidence interval |  | 98.16, 103.0 |  | 96.73, 98.69 |
|  |  | Adjusted treatment mean difference control versus vaccine | | |  |  |
|  |  | Difference |  | 2.919 |  |  |
|  |  | Standard error |  | 1.35 |  |  |
|  |  | p-value |  | 0.314 |  |  |
| D387 |  | Number of animals with observations |  | 15 |  | 16 |
|  |  | Adjusted treatment mean |  | 95.88 |  | 98.74 |
|  |  | Standard error |  | 1.27 |  | 1.83 |
|  |  | 95% confidence interval |  | 93.38, 98.38 |  | 95.12, 102.3 |
|  |  | Adjusted treatment mean difference control versus vaccine | | |  |  |
|  |  | Difference |  | -2.858 |  |  |
|  |  | Standard error |  | 2.23 |  |  |
|  |  | p-value |  | 0.2018 |  |  |
| D570 |  | Number of animals with observations |  | 13 |  | 14 |
|  |  | Adjusted treatment mean |  | 97.65 |  | 96.75 |
|  |  | Standard error |  | 1.22 |  | 1.09 |
|  |  | 95% confidence interval |  | 95.24, 100.0 |  | 94.59, 98.91 |
|  |  | Adjusted treatment mean difference control versus vaccine | | |  |  |
|  |  | Difference |  | 0.893 |  |  |
|  |  | Standard error |  | 1.64 |  |  |
|  |  | p-value |  | 0.5864 |  |  |
| D591 |  | Number of animals with observations |  | 13 |  | 14 |
|  |  | Adjusted treatment mean |  | 95.67 |  | 96.52 |
|  |  | Standard error |  | 0.71 |  | 0.96 |
|  |  | 95% confidence interval |  | 94.26, 97.06 |  | 94.62, 98.42 |
|  |  | Adjusted treatment mean difference control versus vaccine | | |  |  |
|  |  | Difference |  | -0.858 |  |  |
|  |  | Standard error |  | 1.19 |  |  |
|  |  | p-value |  | 0.4725 |  |  |

* The missing samples from day 0 to day 591 correspond to loss to follow-up animals, such as reproductive, low production, lameness, mastitis and accident reasons.
